# The Rwanda Field Epidemiology and Laboratory Training Program: training skilled disease detectives

**Published:** 2011-12-14

**Authors:** Isaac Ntahobakurira, Simon Antara, Tura Boru Galgalo, Jean Baptiste Kakoma, Corine Karema, Thierry Nyatanyi, Rutagwenda Theogene, Odette Mukabayire, David Lowrance, Pratima Raghunathan, Nicholas Ayebazibwe, David Mukanga, Peter Nsubuga, Agnes Binagwaho

**Affiliations:** 1 National University of Rwanda School of Public Health, Kigali, Rwanda; 2 Rwanda Field Epidemiology and Laboratory Training Program, Kigali, Rwanda; 3 Centers for Disease Control and Prevention, Center for Global Health, Division of Global HIV/AIDS, Atlanta, Georgia, USA; 4 Rwanda Ministry of Health, Kigali, Rwanda; 5 Rwanda Animal Resources Development Authority, Kigali, Rwanda; 6 African Field Epidemiology Network, Kampala, Uganda; 7 Centers for Disease Control and Prevention, Center for Global Health, Division of Public Health Systems and Workforce Development, Atlanta, Georgia, USA

**Keywords:** Rwanda, field epidemiology, outbreak investigation, FELTPS, public health

## Abstract

Rwanda still suffers from communicable diseases which frequently lead to epidemics. In addition to other health workforce needs, Rwanda also lacks a public health workforce that can operate multi-disease surveillance and response systems at the national and sub-national levels.In 2009 and 2010 the Rwanda Ministry of Health and its partners from the Government of Rwanda (GOR) as well as the United States (US) Centers for Disease Control and Prevention, the African Field Epidemiology Network, and other partners embarked on a series of activities to develop a public health workforce that would be trained to operate disease surveillance and response systems at the national and district levels. The Rwanda Field Epidemiology and Laboratory Training Program (RFELTP) is a 2-year public health leadership development training program that provides applied epidemiology and public health laboratory training while the trainees provide public health service to the Ministry of Health. RFELTP is hosted at the National University of Rwanda School of Public Health for the didactic training. RFELTP is funded by GOR, the US Presidents Emergency Plan for AIDS Relief and the World Bank; it is managed by a multi-sectoral steering committee headed by the Minister of Health. The first RFELTP cohort has 15 residents who were recruited from key health programs in GOR. Over the first year of implementation, these 15 residents have conducted a variety of field investigations and responded to several outbreaks. RFELTP has also trained 145 frontline health workers through its two-week applied short courses. In the future, RFELTP plans to develop a veterinary track to address public health issues at the animal-human interface.

## Introduction

Despite the numerous public health challenges confronting Sub-Saharan Africa, there is limited spending on health [1]. The low level of investment in health is reflected in the low numbers and sometimes absolute lack of certain cadres of health professionals. Indeed, the lack of health personnel is the main constraint to mobilising responses to health challenges [2]. Various reforms in the health sector have not fully addressed the necessary human infrastructure [3]. Building a public health system that is widely accessible, sustainable, and adaptable to emerging health threats has resulted in improved public health in developed countries. In low-income countries, however, these efforts have stalled due, in part, to the lack of well-trained field epidemiologists and public health laboratorians [4,5].

In Rwanda, the principal causes of morbidity and mortality remain communicable diseases [6]. HIV and malaria place the greatest burden on the health system. The prevalence of HIV infection among the adult population is estimated at 3.0% [7] and the malaria morbidity rate is 18% in the general population (it is 11% in children 8]. Other major causes of morbidity include acute respiratory infections, diarrheal diseases, tuberculosis and malnutrition. Rwanda is also frequently confronted with epidemics of cholera, measles, bacillary dysentery, and typhus [9]. Rwanda, like many African countries, does not have sufficient numbers of trained health staff. Most districts have only seven doctors per 100 000 people [7] which is well below the WHO recommended minimum of 10 doctors per 100 000. Similarly the numbers of other cadres of health workers such as nurses, clinical officers, epidemiologists and laboratory staff are also low.

A collaborative rapid needs assessment done in 2009 in partnership by the Ministry of Health (MOH) and various stakeholders revealed that the capacity of health professionals to manage epidemics was inadequate both at central and district levels. The health system's capacity to respond appropriately to the threat of emerging and re-emerging diseases was low and few outbreaks were detected and reported. Laboratory services to identify infections were routinely unavailable, and investigators frequently neglected the importance of laboratory confirmation. Public health laboratory professionals also lacked key skills in epidemiology to support field investigations. Therefore, for the majority of individuals who became ill or died, the causes of death were largely uninvestigated.

To address this major gap in the public health system in Rwanda, the Rwanda Field Epidemiology and Laboratory Training Program (RFELTP) was established. Specifically, the program was established to build capacity to improve disease surveillance, respond to public health emergencies, and provide information for evidence-based decision making to mitigate the health and economic impact of epidemics and other public health events. Also, there was a desire to bridge the traditional divide between field epidemiologists and public health laboratory scientists, as well as to empower laboratories to effectively lead and manage laboratory services within the public health system. For these reasons, the Government of Rwanda (GOR) and its partners adopted the Field Epidemiology and Laboratory Training Program (FELTP) model of training and established the RFELTP. FELTPs are applied epidemiology training programs that develop a public health workforce that can operate multiple surveillance and response systems and recommend interventions to improve public health [8,9].

### Description of the program

The RFELTP is a 2-year training program in field epidemiology and public health laboratory practice. Established in 2010, the program is a collaboration among MOH, Ministry of Agriculture and Animals Resources (MoAA), National University of Rwanda School of Public Health (NURSPH), Treatment and Research in HIV/AIDS and other Epidemic Infectious Diseases (TRACPlus), the National Reference Laboratory (NRL), the Rwanda Animal Resources Development Authority (RARDA), the African Field Epidemiology Network (AFENET) and the United States (US) Centers for Disease Control and Prevention (CDC). The program has field epidemiology and laboratory tracks. The goals of the RFELTP are to develop self-sustaining, institutionalized capacity to train public health leaders in field epidemiology and field-oriented public health laboratory practice and to provide epidemiological services to the public health system at national, district and local levels. The program is currently hosted in NURSPH, located in Kigali. RFELTP is integrated with other NURSPH postgraduate programs, follows the university calendar, and utilizes subject matter experts from within the NURSPH, CDC, AFENET, MOH and others institutions as needed.

RFELTP targets graduate health professionals working within GOR institutions. Eligibility criteria for admission includes: a minimum of a bachelor's degree in a relevant field of study such as medicine, veterinary medicine, biomedical sciences, biostatistics, or clinical psychology; 3 years of professional experience in a health-related area; Rwandan citizenship; being an employee of GOR; and a letter of recommendation from an employer. For the first cohort, the selection was done by a panel comprising members of partner institutions. Validation of the process was done by the Quality Assurance Directorate of the National University of Rwanda.

In terms of governance, the ultimate decision-making of RFELTP is the steering committee with membership from MOH, TRACPlus, NRL, RARDA, NURSPH, AFENET, CDC and WHO. The committee is currently chaired by the Minister of Health and meets regularly to make decisions and review progress of the program. The program has an Epidemiology and a Laboratory Resident Advisor who provide technical expertise for program implementation.

Residents spend approximately 25% of their time in class for the didactic sessions and 75% in the field. The didactic component comprises five courses interspersed with field placements over the 2 years of the training. The laboratory and epidemiology tracks share 60% of the five courses. Residents begin with an intensive 6 week introductory course that covers basic concepts of biostatistics, surveillance, field epidemiology and public health laboratory techniques, followed by one-to-two-week short courses that cover other course content over the period of two years. During field placements, trainees are assigned to field sites within GOR where they carry out important public health activities, including a surveillance system evaluation, dataset analysis, and outbreak investigations. For the first cohort of 15 residents, all field placement sites were at national-level programs, where residents provide services to all levels of the health system. However, future cohorts will spend their first-year field placement at the national level, while the second-year field placement will be at the district level.

To create a critical mass of public health professionals at all levels of the health system with skills in field investigations, since 2009, the program has offered short courses on outbreak detection, investigation and response to district surveillance officers and laboratorians. So far, a total of 145 persons have been trained an average of three persons from every district. These short courses consist of 2 weeks of classroom teaching and three months of supervised field work, where the participants conduct applied learning projects (e.g., outbreak investigations data analyses) that enable them to become proficient. Short-course participants conduct their field work at their respective work station. This training has lead to an improvement in the timeliness and completeness of reporting of integrated disease surveillance and response (IDSR) priority diseases at district level.

### Key achievements of the program

Since its inception in May 2010 with 15 residents (Table 1), the program has already made positive public health impact in Rwanda (Table 2). The second cohort of 15 residents will begin the program in October 2011.

**Table 1:** Sex and Professional Backgrounds of the First Cohort of Rwanda Field Epidemiology and Laboratory Training Program (RFELTP) residents

Institution of Origin	Number of residents	Professional background	Gender	Track		
			Male	Female	Epidemiology	Laboratory
TRACPlus	7	Six medical doctors and one biostatistician	4	3	7	0
National Reference Laboratory	3	Three biomedical scientists	2	1	0	3
Rwanda Animal Resources Development Authority	3	Three veterinary doctors	3	0	2	1
Rwanda National Police	2	One medical doctor and one clinical psychology	2	0	2	0
**Total**	15		11	4	11	4

**Table 2:** Summary of key achievements of the Rwanda Field Epidemiology and Laboratory Training Program, May 2010 to August 2011

Achievements	Number
Outbreak investigations conducted with appropriate public health response	15
Surveillance systems evaluated	10
Research studies in progress (residents’ thesis)	15
Evaluations (Program or Project)	10
Scientific presentations at conferences	6
Publications by the FELTP residents	3
Short courses conducted	4

All 15 residents currently in the program are employed in the health system at the national level where they are able to apply the skills acquired while in training. Their response to a number of outbreaks in the country has also demonstrated the usefulness of the program (Table 3).

**Table 3:** Outbreaks investigated by Rwanda Field Epidemiology and Laboratory Training Program (RFELTP) Residents from May, 2010 to August 2011

Period	Disease/condition involved	Location	Public health action taken
Jun 2010	1.Cholera outbreak	Nkombo Island, Rusizi District, Western Province	Water chlorination Hand washing and general hygiene education
	2. Suspected malaria outbreak in Bukora Health Centre catchment area	Bukora, Kirehe District, Eastern province	Review of diagnostic criteria
	3.Measles outbreak in Bugarama	Bugarama Sector, Rusizi District, Western Province	Vaccination campaign targeting children under14 years
Nov 2010	4.Measles outbreak in Ruheru	Nyaruguru District, Southern Province	Vaccination campaign
Nov 2010	5. Measles outbreak in Kigali Central Prison	Nyarugenge District, Kigali City	Vaccination and Vitamin A distribution
Aug 2010	6.Typhus fever outbreak in Muhanga prison	Muhanga District Southern Province	Treatment and health education on Hygiene for prisoners
Mar 2011	7.Typhoid fever outbreak in Mukinga Primary School	Kamonyi District, Southern Province	Hygiene education and for the school cooks
Apr 2011	8.Acute Respiratory Illness in Calcutta Orphan Centre	Nyarugenge District, Kigali City	Health education and case management
May 2011	9. Influenza Outbreak in Mpanga Prison	Nyanza District, Southern Province	Health education and case management
May 2011	10. Influenza outbreak in Miyove prison	Gicumbi District, Northern Province	Health education and case management
May-June 2011	11. Measles outbreak Gisagara	Gisagara District, Southern Province	Vaccination campaign
May 2011	12 Suspect Ebola case investigation in Nyamata	Bugesera District, Eastern Province	Health education and re-assurance
Jun 2011	13. Food borne outbreak in Gahini Secondary School	Kayonza District, Eastern Province	Food safety education for students and cookers
	14. Food borne outbreak in Rukara College	Kayonza District, Eastern Province	Food safety education for students and cookers
	15.Typhoid in Nyakinama	Musanze District, Northern Province	Water chlorination and health education on sanitation

The residents have also been actively involved in various disease surveillance activities, including: review of IDSR guidelines, needs assessments, influenza sentinel surveillance, and development of a country epidemic preparedness plan.

The work done by residents has been, and will continue to be, disseminated both locally and internationally at various seminars and conferences. Three were accepted at two different international scientific conferences and plans are ongoing to publish the work of residents in health bulletins and peer-reviewed journals (Table 4).

**Table 4:** Publications by Rwanda Field Epidemiology and Laboratory Training Program (RFELTP) residents submitted and accepted to international scientific conferences from 2009 to 2011

Title	Name of International Conference	Follow up
Cholera Outbreak Investigation in Nkombo Sector of Rusizi District, June 2010	6th TEPHINET Scientific Conference in Cape Town	Accepted in 2010
Measles Outbreak Investigation, Bugarama sector in Rusizi District, Western Province, June 2010	6th TEPHINET Scientific Conference in Cape Town	Accepted in 2010
HIV/AIDS in Kanombe Military Hospital	3rd AFENET Scientific Conference in Mombasa	Accepted in 2009
The Role of Rapid Diagnostic Tests in the Diagnosis of Malaria at Sites of Varying Transmission Intensities in Rwanda, 2010	4th AFENET Conference in Dar -es Salaam, December 2011	Abstract Submitted
Avian Influenza Preparedness in Rwanda in 2006	4th AFENET Conference in Dar- es Salaam, December 2011	Abstract Submitted
Control of Multi-Drug Resistant Tuberculosis in Rwanda 2005-2010	4th AFENET Conference in Dar- es Salaam, December 2011	Abstract Submitted
Evolution of CD4+T-Cell Counts in a Cohort of Patients on Anti-Retroviral Therapy in Rwanda, 2010	4th AFENET Conference in Dar -es Salaam, December 2011	Abstract Submitted
Evaluation of the Meteorological Surveillance System in Rwanda, 2011	4th AFENET Conference in Dar- es Salaam, December 2011	Abstract Submitted
Fine Needle Aspirate as a Useful Tool in the Diagnosis of Tuberculous Lymphadenitis	4th AFENET Conference in Dar- es Salaam, December 2011	Abstract Submitted
Free Rabies Vaccination Program Improved Dog Management Skills among Dog Owners in Rwanda, 2006-2009	4th AFENET Conference in Dar -es Salaam, December 2011	Abstract Submitted
Evaluation of the Measles Surveillance System in Rwanda, 2010	4th AFENET Conference in Dar- es Salaam, December 2011	Abstract Submitted
Comparative Evaluation of Two Rapid Field Tests for Malaria Diagnosis in Rwanda, January 2010	4th AFENET Conference in Dar- es Salaam, December 2011	Abstract Submitted
Epidemic Typhus Outbreak in a Rwandan Prison, 2010	4th AFENET Conference in Dar- es Salaam, December 2011	Abstract Submitted
Botulism Outbreak among Prisoners in Rwanda, 2009	4th AFENET Conference in Dar -es Salaam, December 2011	Abstract Submitted
Kigali City Solid Wastes Management: A Case-Study of Nyanza Open Landfill Final Disposal Site- Rwanda, 2010	4th AFENET Conference in Dar -es Salaam, December 2011	Abstract Submitted
Comparative Evaluation of Two Rapid Field Tests for Malaria Diagnosis in Rwanda, January 2010	4th AFENET Conference in Dar -es Salaam, December 2011	Abstract Submitted
Evaluation of the Tuberculosis Surveillance System in Rwanda, 2010	4th AFENET Conference in Dar es Salaam, December 2011	Abstract Submitted
Evaluation of Poliomyelitis Surveillance System in Rwanda, 2010	4th AFENET Conference in Dar-es Salaam, December 2011	Abstract Submitted

The RFELTP has been primarily funded by the US President's Emergency Plan for AIDS Relief through CDC; however, the program has also secured some resources through the World Bank's East African Public Health Laboratory Network Project and intends to seek other funding sources. Moreover, the MOH has included the program in the National Human Resources for Health Strategic Plan, 2011-2016 and aims to ensure that the program is sustainable when donor funding ceases.

Currently, the program includes two tracks in field epidemiology and laboratory. There are plans to add a veterinary track in the spirit of a “One Health approach” in which human health, animal health, wildlife and disaster management professionals will be trained to work as one team. RFELTP also plans to expand to include residents from district hospitals in the third and subsequent cohorts to strengthen skills at decentralized levels of the health sector in order to improve timely outbreak detection and response and effective public health surveillance system and management at lowest level of the health system.

The program intends to utilize graduates of the first cohort as field mentors and instructors. This initiative should ensure that subsequent cohorts will have adequate supervision and support while in the field.

## Challenges

The RFELTP still has a shortage of local teaching staff, field site mentors and supervisors. This staff shortage is especially acute during supervision of field work. Current residents, by virtue of their positions at the national level, have to do their jobs as well as fulfil the requirements of the program. This strains the residents and creates a challenge to the completion of field work, however it is a short term challenge as the program progresses, the graduates will be able to work more effectively in their jobs and it will also allow for more people to be trained. Sustainability of funding will continue to be challenge, the program will have to look for efficiencies to reduce costs as well as diversify the funding base.

## Conclusion

The Rwanda FELTP has successfully been implemented for one year. Despite its short period of existence, the program has responded to several critical needs of the country as seen in the number of outbreaks investigated and surveillance systems evaluated. The demand for the program has increased gauging from the increased number of applications for cohort two due to start later this year. The knowledge and skills acquired by residents of the program will be invaluable to the country and contribute immensely to improved implementation of health programs in the country. Given the successful launch of the RFELTP and the achievements so far, we recommend that the various partners and the GOR engage in finding ways of sustaining it including developing specific budget lines in their annual budgets.
